# Occurrence, Role, and Challenges of MicroRNA in Human Breast Milk: A Scoping Review

**DOI:** 10.3390/biomedicines11020248

**Published:** 2023-01-18

**Authors:** Adrianna Kondracka, Paulina Gil-Kulik, Bartosz Kondracki, Karolina Frąszczak, Anna Oniszczuk, Magda Rybak-Krzyszkowska, Jakub Staniczek, Anna Kwaśniewska, Janusz Kocki

**Affiliations:** 1 Department of Obstetrics and Pathology of Pregnancy, Medical University of Lublin, 20-059 Lublin, Poland; 2 Department of Clinical Genetics, Medical University of Lublin, 11 Radziwillowska Str., 20-080 Lublin, Poland; 3 Department of Cardiology, Medical University of Lublin, 20-059 Lublin, Poland; 4 Department of Oncological Gynecology and Gynecology, Medical University of Lublin, 20-059 Lublin, Poland; 5 Department of Inorganic Chemistry, Medical University of Lublin, 20-059 Lublin, Poland; 6 Department of Obstetrics and Perinatology, University Hospital, 30-688 Krakow, Poland; 7 Department of Gynecology, Obstetrics and Gynecologic Oncology, Medical University of Silesia, 40-055 Katowice, Poland

**Keywords:** microRNA, human breast milk, polymerase chain reaction (PCR), microRNA characterization

## Abstract

MicroRNAs are non-coding segments of RNA involved in the epigenetic modulation of various biological processes. Their occurrence in biological fluids, such as blood, saliva, tears, and breast milk, has drawn attention to their potential influence on health and disease development. Hundreds of microRNAs have been isolated from breast milk, yet the evidence on their function remains inconsistent and inconclusive. The rationale for the current scoping review is to map the evidence on the occurrence, characterization techniques, and functional roles of microRNAs in breast milk. The review of the sources of this evidence highlights the need to address methodological challenges to achieve future advances in understanding microRNAs in breast milk, particularly their role in conditions such as neoplasms. Nonetheless, remarkable progress has been made in characterizing the microRNA profiles of human breast milk.

## 1. Introduction

It has been shown that breastfeeding brings multiple nutritional, developmental, immunological, and biological benefits to infants, making breast milk the most appropriate food for newborns. Numerous studies have shown a positive association between the duration of breastfeeding and variables such as intelligence quotient, income, and educational performance. In addition, the physical health benefits of breastfeeding include a lower incidence of childhood illnesses, such as gastroenteritis, pneumonia, and asthma. The World Health Organization recommends that infants should be exclusively breastfed for about the first six months of their life [1]. Apart from demonstrating the advantages of breast milk, research on the subject has also focused on identifying the mechanisms underlying these characteristics. As a result, some features of breastfeeding have been explained by the identification of molecules and other factors present in breast milk that confer certain characteristics. For instance, the presence of secretory IgA and lactoferrin has been linked to the immunogenic potential of human breastmilk, although the specific mechanism of their transfer to the infant remains poorly understood [2]. Recent studies have reported that human milk contains factors such as non-coding RNA species that are capable of regulating the genetic development of infants’ biological systems [2].

MicroRNAs (miRNAs) are a group of regulatory RNA species capable of modulating the activation of certain mRNA targets to influence a wide range of biological processes. Segments of mRNA consist of short nucleotide sequences, between 19 and 24, and are derived from hairpin precursors [3]. The biogenesis of miRNAs is controlled by RNA polymerase II to generate primary microRNAs, which are modified by various proteins from the RNAse III family to produce shorter microRNAs. MicroRNAs are known to be ubiquitous, occurring in multiple tissue types, cells, and such biological fluids as blood, saliva, and tears, as well as breast milk. The molecules regulate many processes, including organogenesis, immune activity, and cellular metabolism, as well as cell differentiation [2]. Recently the presence of other small RNA species in body fluids has been reported, including circular RNAs (circRNAS), long non-coding RNAs, transfer RNAs (tRNAs), and small nuclear RNAs (snRNAs) [3,4]. The role of these non-coding RNA species in breast milk and other fluids remains poorly understood and is the subject of ongoing research on human breast milk. The current research on the subject has highlighted the role of breast milk microRNAs in infant biological system development. While more roles of microRNAs are still being discovered, the most established role is the regulation of specific genes involved in infant immune system development [4]. As a result of their expression in multiple body fluids and involvement in various biological processes, micro-RNAs have the potential to be used as non-invasive biomarkers for monitoring diseases in which their levels change.

Multiple studies have been published on breast milk characteristics and composition, including the occurrence of non-coding RNAs in animal and human breast milk [1,2,4]. These studies cover a wide range of subjects, from the concentration of specific RNA species to the function of these molecules in breast milk. The most commonly reported role of microRNAs in human breast milk is immune modulation. The potential for these molecules to play a role in the immune system development pathways in infants has been considered. Despite these recent advances, the extent and types of sources of evidence for the occurrence and role of microRNAs in human breast milk remain limited [4]. Therefore, a scoping review is necessary to map the scholarly studies on the subject to identify emerging evidence sources and novel methodological approaches to the study of the subject. The scoping review undertaken here aims to map the literature on this subject and highlight potential research gaps and future trends in its scholarly investigation. The following research questions were formulated to guide the scoping review:

1. What microRNA subtypes occur in human breast milk and what are their functions?
2. What conditions affect the quantities and types of microRNA in breast milk?

## 2. Methods

### 2.1. Protocol

While a formal review protocol was not published a priori in this study, the methodological approach was informed by the Preferred Reporting Items for Systematic Reviews and Meta-analysis Protocols (PRISMA-P), as well as scoping review guidelines from the Joanna Briggs Institute. The reviewer revised and adapted the protocol components to align with the review aims and based the report items on the Preferred Reporting Items for Systematic reviews and Meta-Analyses extension for Scoping Reviews (PRISMA-ScR) Checklist, as well as its elaboration by Tricco et al. [5].

### 2.2. Data Sources

A comprehensive electronic search of the following databases to identify sources of evidence on the subject was conducted by the reviewer: PubMed (Medline), The Cochrane Library, Web of Science, and Scopus. No additional sites were searched for unpublished sources or grey literature due to the focus on peer-reviewed, methodologically rigorous sources of evidence. The search strategy involved various combinations of MeSH terms and Boolean operators combining multiple formats and synonyms of two principal concepts: microRNA and breast milk. The specific keywords used to retrieve sources from the databases were microRNA, miRNA, human breast milk, breastfeeding, and lactation. each concept was expressed using multiple synonyms and abbreviations, which were operationalized using the Boolean operators OR and AND, to ensure that all relevant sources were retrieved from the target databases. This search strategy was slightly adapted to comply with the features offered by each database or optimized to give the widest range of results limited to sources relevant to the topic. The search string used in the PubMed database, for instance, was as follows:

(microRNA OR miRNA OR qRNA or lncRNA) AND (breastmilk OR breast milk OR human breast milk OR human breastmilk OR colostrum OR breastfeeding OR lactation).

### 2.3. Eligibility Criteria

As a scoping review, the study adopted inclusive eligibility criteria to allow the identification of the largest number of relevant sources of evidence on the role of microRNAs in human breast milk. The analysis included the detection, characterization, quantification, and functional analysis of microRNA components in human milk, which enabled retrieval of sources that aligned with the review’s aim of identifying microRNA types, quantities, and functions. The exclusion criteria included papers published in languages other than English, a specification that was necessary to ensure the accuracy of the review by avoiding potential changes due to translation. In addition, narrative review articles, book chapters, and conference papers were excluded from the review to ensure that only high-level primary evidence sources were included.

### 2.4. Source Selection

**Figure 1:**
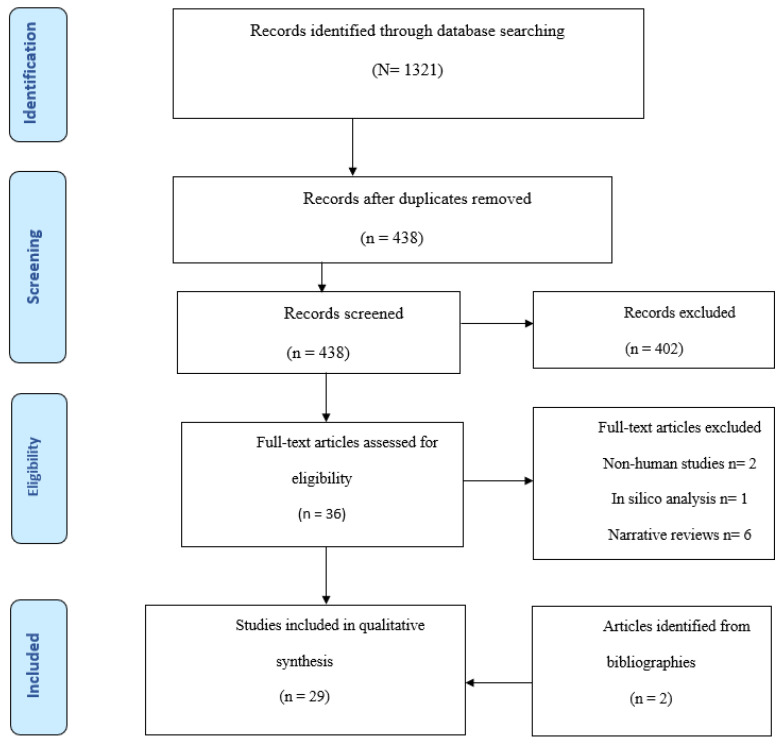
Flowchart: selection of sources.

The retrieved sources were screened by the reviewers in two phases. The first phase involved the removal of duplicates, which was performed using free online software, and then double-checked manually. After duplicate removal, the titles, abstracts and keywords of sources were screened to identify those that mentioned the two main concepts and to eliminate non-English sources. In the second phase, the abstracts of sources retrieved by title were screened to determine the relevance to the review topic and identify the methodology used, particularly whether microRNA analysis was performed. The screening of abstracts also helped the reviewers identify articles whose methodologies aligned with the aims and inclusion criteria of the review. Articles whose methodology was not clearly evident were screened by the reviewer using the Section 2. Finally, the reference sections of the final list of articles were assessed to identify potential sources that could be included in the review (See Figure 1).

### 2.5. Data Extraction and Charting

A standardized data abstraction chart was developed and used for extracting relevant information from the sources. The table included the most relevant information on each study, including the publication year, study location, aims, participants or sample sizes, and findings. These characteristics were also relevant to the subsequent synthesis of results from all included studies (see Table 1).

### 2.6. Synthesis of Results

The results from the included studies were presented in a narrative synthesis, outlining the key types of studies, analysis methods, types of non-coding RNAs in breast milk, functions of microRNAs in milk, as well as future trends. The results synthesis also included the categorization of the goals and a review of the methodological techniques used by the researchers.

## 3. Results

### 3.1. Selection of Sources

The initial database search returned a total of 1321 results, which were filtered in the first phase of the selection strategy involving duplicate removal and exclusion of non-English entries, as well as those dealing with unrelated topics. In the second phase, a total of 36 sources underwent full-text analysis for relevance and methodological rigor, resulting in the exclusion of nine sources, six of which were narrative reviews, six studies on only non-human breast milk, and one in silico analysis. Screening of the reference sections led to the identification of two more sources, the inclusion of which resulted in a total of 29 articles included in the scoping review (Figure 1).

### 3.2. Study Characteristics

The studies included in this review were published between 2010 and 2022, the majority of which were conducted in 2021 (five studies) and 2022 (five studies). The most commonly investigated topic was the microRNA profile of human breast milk, using an explorative approach to determine what types, concentrations, and functional categories of microRNAs and other non-coding RNAs are found in human milk [8,11,12,14,30] (See Table 1). As shown in Table 1, the studies on the subject can be categorized into five groups based on the research focus. Studies that focused on microRNA extraction techniques highlighted the methodological approaches used in collecting and processing breast milk for microRNA analysis, isolating the exosomes, and quantifying the microRNA content. The methodologies used for microRNA quantification are an important aspect of the subject as they appear to account for a significant number of inconsistencies in findings. Importantly, as noted by Ahlberg (2021), standardization of quantification techniques is a priority for research on the subject, the success of which has implications for the potential future use of microRNAs as disease markers. The second category of studies focused on the types and quantities of microRNAs in milk. A review of findings from these studies confirmed that a large variety of microRNA subtypes occur in breast milk, with their quantities varying unpredictably and their role remaining poorly understood. The most commonly reported microRNAs, however, occur quite consistently, regardless of the processing techniques or time during the lactation period. These include miR-148a-3p, miR-30b-5p, and miR-200a. The third category of studies focused on how breast milk processing techniques, such as pasteurization, time during lactation, environmental conditions, including temperature, maternal characteristics, such as obesity, and infant maturity, affect the type and amounts of microRNAs isolated from milk. Here, the most significant results were that microRNA types and quantities vary during the lactation period, depend on the source of milk (breast milk versus infant formulas), the mother’s weight, and on pasteurization, which reduces the microRNA content in breast milk. The fourth research category explored the role of microRNAs in disease, which was perhaps the least researched aspect of the subject. Nonetheless, the studies in this group showed that breast milk microRNAs play a role in the development of neonatal jaundice and necrotizing enterocolitis and serve as a potential biomarker in breast cancer. The fifth category of publications focused on the physiological functions of microRNAs in breast milk. It has long been apparent that these RNA species play an important role in the regulation of genetic development in infants. The most prominent role of microRNAs is the regulation of gene expression in the development of the immune system in infants, although the specific genes involved are poorly characterized. In addition, microRNAs in breast milk perform epigenetic modulation of certain genes involved in infant brain development, physical cellular development, and endocrine system development. Importantly, studies have demonstrated that microRNAs may be transferred to the infant through intestinal absorption due to the species’ hardiness and ability to resist digestion (Table 1).

### 3.3. MicroRNA Isolation and Quantification

Various methodological techniques were applied in different studies depending on the aims. Techniques, such as centrifugation and fractionation, were described or referred to in most sources, as most studies focused on isolating microRNAs from milk samples. In addition, microRNA detection was conducted using two closely related approaches, real-time PCR [17,18,28,30,31] and deep sequence analysis [12,14,26]. A number of expression kits were described, but the TaqMan miRNA kit was the most popular reverse transcription kit used in the studies [6]. 

### 3.4. Types and Functions of microRNAs

While most researchers focused exclusively on human breast milk, a few performed comparative analyses of microRNA profiles in other species, including cows and goats [25,28]. The concentration and types of microRNAs isolated from breast milk varied widely, yet several species were identified as the most abundant. These included miR-148a-3p, miR-30b-5p, and miR-200a [15,23,26,31]. Similarly, researchers attributed various functions to the identified microRNAs, ranging from regulation of genes that control immune function [2,3,20,25,28] to epigenetic modulation of genes that determine physical health and infant neurodevelopment [29,30,31] (Table 1).

## 4. Discussion

MicroRNAs are small gene segments involved in the epigenetic regulation of multiple pathways. Their presence in several biofluids has been demonstrated, including in plasma and, as the abovementioned studies showed, in breast milk. Quantifying and characterizing microRNAs and other non-coding exons have attracted researchers’ attention because of their potential application in the detection, prevention, and treatment of health disorders, including nutritional conditions and neoplasms. MicroRNAs in breast milk appear to have specific functions, most notably to modulate the development of the infant immune system. Breastmilk provides a unique and convenient pathway for microRNAs to be transferred to the infant, where they appear to be absorbed in the intestinal mucosa and where their epigenetic modifying effects are employed. Importantly, the available literature does not provide evidence on the specific cellular targets and the mechanisms involved in epigenetic modulation in relation to the immune system. Similarly, the role of microRNAs in neurodevelopment, which is relevant to the functional maturation of this system in newborns, has been suggested in the literature, while its exact mechanisms remain relatively unexplored.

The literature provides insights not only into the functional implication of microRNAs, but also highlights the variety of methodological approaches used to conduct the isolation and characterization of the molecules. The typical process of isolating breast milk microRNAs includes collecting and storing milk samples, centrifugation, fractionation, and selection of a target phase, as well as molecule isolation, using methods such as reverse transcription and quantification. Different types of equipment and methodological specifications are used to conduct each of these processes, but there is no standardized approach or choice of tools to ensure uniformity. Methodological approaches to the isolation and quantification of microRNAs are, however, somewhat limited. In the current studies considered, the segments are typically identified in a three-step process involving extraction through sample centrifugation and fraction, reverse transcription, and quantification [3,18,31]. Several methods are used for microRNA quantification, the most common being quantitative PCR [3,18,28,31]. Microarray techniques [1,7] and small RNA sequencing [12,14,16] enable the processing of large amounts of genetic material at the same time. The development of deep sequencing techniques has enhanced the sensitivity of gene-detection systems, enabling the identification of minute amounts of material in samples. The performance of each of these methods, and how it varies depending on the sample source and processing method, are important considerations in order to standardize methodological approaches to microRNA characterization. Currently, practical microRNA characterization relies on a combination of different methods and tools. Standardized identification and quantification are crucial to assign therapeutic target and pathologic marker roles to unique microRNA molecules. 

An important aspect of the empirical research on this subject is to identify the specific microRNAs that consistently occur in breast milk and to quantify them in order to build a profile of normal breast milk microRNA content. Unfortunately, this goal is not easy to achieve since a large number of microRNA subtypes occur in breast milk and some of them are not as predictable as others. Various concentrations of microRNAs reported in different studies appear to be even more challenging, probably due to the use of differing methodologies. At this point, a wide range of microRNAs have been found in human breast milk. While different researchers typically isolate various microRNA species, a few segments are consistently present in breast milk, including miR-148a-3p, miR-30b-5p, and miR-200a [1,13,23,26].

Logically, the next step in the investigation is to determine the role of these commonly occurring microRNAs, which can be challenging. Determining the functional role of microRNAs in breast milk relies on the identification of known microRNAs in the biofluids to provide insight into the systems targeted by the microRNAs. As indicated by multiple researchers, the functions performed by microRNAs in breast milk are similarly heterogenous, with the best characterized role being the regulation of genes involved in infant immune system development [2,3,20,25,28]. In addition, researchers have suggested that microRNAs in breast milk are involved in regulating genes responsible for neurodevelopment [30], endocrine function, and protection from such diseases as necrotizing enterocolitis [22] and atopic dermatitis [24]. Interestingly, microRNAs have been found to cause some diseases too, including breast milk jaundice [21] and breast carcinoma [23]. While further research is needed to confirm the role of microRNAs in the development of these conditions, it is already known that these segments have the potential to be used as biomarkers and treatment targets in patients.

One area of research that needs additional investigation is the association between microRNA expression and various maternal and environmental characteristics. A significant number of studies have already been conducted to evaluate the impact of maternal diet, weight, BMI, post-partum period, age, and other factors on the amount and types of microRNA expressed in milk [9,10,14,18,25,30]. However, the findings from such investigations showed inconsistencies in factors found to impact microRNA expression. Similarly, the mechanism of transfer of microRNAs to infants needs to be further investigated; a growing number of studies suggest intestinal observation of unaltered segments due to high resistance to hostile conditions [2,19,26,27]. Overall, many studies have been conducted on breast milk microRNA, providing a considerable amount of evidence on previously unclear mechanisms. However, much research still needs to be undertaken before practically useful evidence is found. A notable limitation of the published studies was small sample sizes and the lack of a standardized approach to microRNA extraction and quantification.

## 5. Conclusions

The potential applications of microRNAs in preventive medicine and treatment have driven extensive research on their occurrence and role in various biofluids. The current scoping review has highlighted the identification of an increasing number of microRNAs in breast milk. Their isolation and comparison with known functional microRNAs have enabled researchers to speculate on what roles they may play in the infant and in the lactating mother. The best understood functional role of breastmilk microRNAs is the regulation of the development of the infant immune system, which is supported by the occurrence of specific types of microRNAs known to be involved in immune system development and regulation. By searching for specific microRNAs with known functions, researchers have also identified the role of the molecules in neurodevelopment. Interestingly, the expression of microRNAs in breast milk has been shown to be associated with certain diseases occurring in infants and mothers, including breast neoplasms and neonatal jaundice. While the evidence for this association is uncertain, it represents a potential first step towards the use of the molecules in disease monitoring. The methodological techniques applied in the isolation, characterization, and functional analysis of microRNAs in biofluids have important implications for the results obtained. Among the studies reviewed, various techniques were applied in the processing, fractionation, and quantification of microRNAs, pointing to possible challenges in standardizing the detection and analysis of these molecules. Future research should be targeted at investigating the pathophysiological bases of the different functions of microRNAs in breast milk, as well as developing and adopting standard isolation and characterization methodologies.

## Figures and Tables

**Table 1:** Summary of studies.

First Author, Year (Country)	Study Aims	Methods	Sample Size	Findings
**1. microRNA EXTRACTION TECHNIQUES**				
Ahlberg, 2021 (Sweden) [6]	To determine the efficiency of five RNA extraction kits in retrieving microRNA from human breast milk.	Five internationally available RNA extraction kits were used to retrieve the microRNA hsa-miR-39-3p and an exogenous control microRNA from four samples of skim milk. Total and specific RNA extracts were quantified using a Qubit microRNA assay kit, and performance was compared using statistical analysis in GraphPad Prism.	Five international kits were compared to determine the extraction efficiency: Promega, Zymo, Norgen RNA, Norgen Cell, and Sigma-Aldrich.	Two of the extraction kits, Promega and Zymo, had the highest yields of mRNA extracts. Variations in kit performance highlight the need for a standardized protocol for research on mRNA in milk.
Alsaweed, 2015 (Australia) [7]	To standardize microRNA isolation from three fractions of centrifuged human milk using different methods.	Human milk samples were fractionated, and microRNA content was extracted from each layer (lipid, skim milk and cells) using 8 commercially available kits.	49 milk samples from 29 lactating women.	All three layers of fractionated milk had microRNA content, with the cellular and lipid layers having the highest concentrations.
**2. TYPES AND QUANTITIES OF ISOLATED microRNAS**				
Alsaweed, 2016 (Australia) [8]	To determine the types of microRNAs present in human milk after different lactation periods.	Milk samples were collected at 2, 4, and 6 months of lactation. Bioinformatics analysis of microRNAs extracted from the lipid and cell layers was conducted to identify known and predicted microRNAs.	Breast milk was obtained from 10 lactating women	MicroRNA content in cell fractions was higher than in lipid fractions. The most common microRNA species from both columns was let-7f-5p. Across the three lactation stages, the profile (amount and species) of microRNA in breast milk changed significantly, suggesting adaptation to infant’s needs.
Bozack, 2020 (USA) [9]	To investigate the association between maternal psychological stress and the microRNA amount and quantity expressed in breast milk.	Psychological stress was assessed using Life Stressor Checklist-Revised, while microRNA profiling was performed using the TaqMan Open Array Human miRNA panel. Statistical analysis based on regression was used to determine association.	Breast milk was obtained from 80 lactating women	744 microRNAs were detected in total. The quantity of microRNA in breast milk was negatively correlated with psychological stress scores. The types of microRNA extracted also differed depending on psychological stress, with six species demonstrating the highest association with high stress scores.
Carney, 2017 (USA) [10]	To determine the correlation between gestational age at delivery and maternal milk microRNA profiles.	MicroRNA content from breast milk from mothers of infants born prematurely was compared with breast milk collected from mothers of term babies.	Colostrum and hind milk samples were obtained from the total of 44 mothers.	The quantities of nine microRNAs differed across the two groups. The affected microRNAs had target genes related to metabolism and lipid formation.
Floris, 2015 (France) [11]	To investigate changes in breast milk microRNA content in a 24-h period.	MicroRNA content from samples collected at different times were analyzed to determine expression of 4 reference species.	84 milk samples were collected at different times from 22 healthy mothers.	Two microRNA species, hsa-miR-16-5p, hsa-miR-21-5p, were stably expressed throughout the day, supporting their potential for use as endogenous reference species in future studies.
Munch, 2013 (USA) [12]	To determine the amount and types of microRNAs in human breast milk.	Deep sequence analysis of human breast milk to identify known and novel microRNAs.	5 lactating women	Generated sequences matched 308 known mature microRNAs targeting 9074 genes; and 21 novel microRNAs. A number of the novel microRNAs had origins from enriched foods.
Rubio, 2018 (Spain) [13]	To characterize the profiles of small RNA species in milk and plasma.	Isolation and quantitation of clusters of small RNAs from plasma and breast milk samples.	15 healthy postpartum mothers	Both milk and plasma expressed microRNAs, piwi-interfering RNAs (piRNAs), tRNAs, and small nucleolar RNAs. The concentrations of different small RNAs varied between the two biofluids.
**3. EFFECTS OF PROCESSING, TIMING, ENVIRONMENTAL, AND MATERNAL CONDITIONS**				
Hicks, 2022 (USA) [14]	To identify the most abundant microRNAs in milk and determine their variation with time.	Deep sequence analysis to identify microRNAs in samples collected at different times.	503 samples obtained from 192 mothers	The quantity of most microRNA species increases with breast milk maturity, with nearly half being influenced by maternal diet.
Leiferman, 2019 (USA) [15]	To assess exosome and microRNA expression in human breast milk and commercial infant formulas.	RNA sequencing of fresh and stored breast milk and infant formulas	Breast milk was obtained from 5 lactating mothers.	Fresh human breast milk had 21 microRNAs stored in exosomes, while infant formulas had none. Storage at 4 °C resulted in a 49% decrease in the microRNA content in breast milk.
Raymond, 2021 (France) [16]	To characterize the microRNA profile of breast milk from week 2 to the second month post-partum	Next generation sequencing of breast milk samples collected at different times.	44 mothers	685 microRNAs were isolated, 35 of which were present in stable quantities throughout the lactation period.
Shiff, 2019 (Israel) [17]	To investigate the differences in the microRNA profiles of breast milk collected from mothers of premature and term infants.	Quantitative real-time PCR detection and comparison of four common microRNA species	38 healthy mothers	MicroRNA 320 occurred more commonly in colostrum of term infant mothers, while microRNA 148a occurred more commonly in premature human breast milk.
Shah, 2021 (USA) [18]	To examine the association between maternal obesity/overweight and expression of specific microRNAs in breast milk.	Real-time quantitative PCR for 6 specific microRNAs, followed by regression analysis to determine association with different maternal characteristics.	60 lactating mothers	Two of the six microRNAs, miR-148a and miR-30b, were lower in overweight mothers than in normal weight mothers.
Smyczynska, 2020 (Poland) [19]	To determine the effects of pasteurization on the microRNA content in human breast milk.	Milk samples were pasteurized using either Holder pasteurization or high-pressure processing, or left unpasteurized. Subsequently, they were analyzed for microRNA content using quantitative sequencing techniques.	Milk samples were collected from 3 volunteers	Pasteurization resulted in significant reduction in microRNA content in the breast milk samples.
Wang, 2022 (USA) [20]	To assess the feasibility of microRNA analysis in frozen human breast milk.	Breast milk samples were frozen at -80^o^ C, then analyzed for microRNAs and exosomes using PCR and immunoblot techniques.	Milk samples were obtained from three mothers of preterm infants.	While freezing resulted in significant reductions in microRNA and exosome levels, analysis was still feasible.
Wu, 2020 (China) [1]	To investigate the expression of microRNA in human colostrum.	Microarray analysis of colostrum samples and prediction of microRNA targets.	18 lactating volunteers	The microRNA composition of human milk changes through the lactation period. 49 microRNAs were consistently found in colostrum in higher concentrations than in milk.
**4. ROLE OF microRNAS IN DISEASES**				
Yang, 2020 (China) [21]	To determine whether microRNAs in breast milk contribute to breast milk jaundice.	Flow cytometry, Western blotting, and nanoparticle tracking were used to monitor exosomes in breast milk, and association analysis was used to find association with breast milk jaundice.	32 mother-infant dyads	Based on the expression of a few microRNA species, the evidence supports a role for microRNAs in breast milk jaundice.
Pisano, 2020 (USA) [22]	To determine whether extracellular vesicles (EVs) from human breast milk can protect against experimentally induced necrotizing (NEC) enterocolitis in mice.	Lactating mice were randomized to either breastfeeding without NEC, NEC with no breastfeeding, NEC with intraperitoneal EVs, and NEC with oral EVs. Histological analysis was done to determine the extent of NEC.	Not indicated	Breastfed rats had 0% NEC, while 62% of those with experimentally induced disease experienced it. Intraperitoneal EV reduced NEC to 29%, while oral administration reduced it to 11.9%.
Gu, 2014 (China) [23]	To determine the association between milk stasis and breast carcinogenesis and the potential role of microRNAs as biomarkers.	Milk samples from patients with milk stasis and patients with both stasis and breast cancer were analyzed for microRNA profiles.	A total of 20 patients	MicroRNA profiles differed between samples from patients with and without a neoplasm. In addition, microRNAs with roles in tumor suppression and oncogenesis had pro-neoplastic expression profiles.
Simpson, 2015 (Norway) [24]	To investigate the potential mediating role of breast milk microRNAs in the anti-dermatitis effects of probiotic supplements.	Small RNA sequencing for microRNAs in breast milk samples, followed by statistical analysis to determine correlations between expression profiles and probiotic effects.	54 lactating mothers	The biological significance of microRNAs in atopic dermatitis was not apparent.
**5. PHYSIOLOGICAL FUNCTIONS OF microRNAS**				
Yun, 2021 (South Korea) [25]	To explore microRNA profiles of human, bovine, and caprine milk.	RNA sequencing of fractionated milk samples.	Milk samples were obtained from 2 human volunteers, 6 cows, and 3 goats.	Several dietary micro-RNAs are expressed in human, cow, and caprine milk. These species are mostly involved in immune regulation.
Zhou, 2012 (China) [26]	To investigate microRNAs in human breast milk.	Deep sequence analysis of samples for microRNA identification.	Breast milk was collected from 4 healthy mothers.	602 unique microRNAs were isolated from the milk samples. Resistance to harsh conditions suggests transfer to infants via the digestive tract.
Kahn, 2018 (USA) [27]	To determine the effect of in vitro digestion on microRNA from breast milk of mothers of preterm infants.	Exosomes from milk samples were isolated and lysed using commercial RNAses, exposed to in vitro digestive enzymes, and subsequently sequenced to determine surviving contents.	Milk samples were collected from 10 mothers.	MicroRNAs that are specific to preterm infant milk were not affected by in vitro digestion, suggesting increased susceptibility of preterm infants to maternal microRNA-driven epigenetic effects.
Na, 2015 (China) [28]	To screen for presence of 5 immune-related microRNAs in human, goat, and cow milk.	Real-time PCR of breast milk samples to identify MiR-146, miR-155, miR-181a, miR-223, and miR-150 species.	Milk samples were collected from 3 human volunteers, 2 lactating goats, and 3 dairy cows.	All five microRNA species were found in human and goat milk, and four were present in cow milk.
Karlsson, 2016 (USA) [29]	Screening for developmentally important lncRNAs in breast milk.	Quantitative analysis was used to determine the presence of specific non-coding RNAs in breast milk samples.	Breast milk was collected from 30 participants.	55 of the 87 non-coding RNAs screened for were found, demonstrating a possible role in childhood development.
Kosaka, 2010 (Japan) [2]	To determine the importance of breast milk microRNAs in infant immune regulation.	MicroRNA content of breast milk was screened for species known to play a role in immune function.	6 breastfeeding women provided milk samples	Breast milk has high levels of immune-regulating microRNAs in the first 6 months of lactation. These microRNAs are stable in acidic conditions, allowing for dietary intake.
Kupsco, 2021 (USA) [30]	To characterize breast milk microRNA in population cohort and evaluate association with maternal characteristics.	Small RNA PCR sequencing of human milk samples to identify the most common microRNA species, followed by association analysis with several maternal characteristics.	Breast milk was obtained from 364 mothers	A total of 1523 microRNAs were detected in at least two samples. Three of the most common clusters demonstrated common ontogeny in pathways enriched by such systems as endocrine development and neurodevelopment. Association of microRNA expression with maternal BMI, time of milk collection, and smoking was demonstrated.
Lukasik, 2017 (Poland) [31]	To screen human breast milk for presence of specific plant-derived microRNAs.	Real-time quantitative reverse transcription PCR of human breast milk samples to detect 5 specific plant-derived microRNAs.	6 lactating mothers	Two of the 5 screened microRNAs were isolated in breast milk samples. These microRNAs are believed to be acquired from ingested plant-based foods, and to have a role in infant development.
Velez-Ixta, 2022 (Mexico) [3]	To assess the expression of immunoregulatory microRNAs in human breast milk.	Expression of 5 specific microRNAs was assessed using quantitative PCR in milk samples and infant formula.	60 lactating mothers	All five screened microRNAs (miR-146b-5p, miR148a-3p, miR155-5p, mir181a-5p, and mir200a-3p) were detected in human breast milk, but only very small concentrations of the species were found in infant formulas.

## Data Availability

Not appliable.
